# Synergistic terahertz platforms for precision oncology

**DOI:** 10.7150/thno.128658

**Published:** 2026-01-14

**Authors:** Yan Chen, Xiaodan Kou, Jing Zou, Peng Chen, Junfeng Liu, Yanru Gu, Min Zhuang, Hongtao Xiao, Yuying Li, Man Lu, Shugang Qin

**Affiliations:** 1 Department of Pharmacy, Sichuan Clinical Research Center for Cancer, Sichuan Cancer Hospital & Institute, Sichuan Cancer Center, University of Electronic Science and Technology of China, Chengdu, 610000, China.; 2 Department of Respiratory Critical Care, The Affiliated Hospital of Southwest Medical University, Luzhou, 646000, China.; 3 Department of Hepatopancreatobiliary Surgery, Sichuan Clinical Research Center for Cancer, Sichuan Cancer Hospital and Institute, Affiliated Cancer Hospital of University of Electronic Science and Technology of China, Chengdu, 610000, China.; 4 Department of Interventional Radiology, Sichuan Clinical Research Center for Cancer, Sichuan Cancer Hospital & Institute, Sichuan Cancer Center, University of Electronic Science and Technology of China, Chengdu, 610000, China.; 5 Department of Ultrasound, Sichuan Clinical Research Center for Cancer, Sichuan Cancer Hospital & Institute, Sichuan Cancer Center, Affiliated Cancer Hospital of University of Electronic Science and Technology of China, Medicine & Laboratory of Transitional Research in Ultrasound Theranostics, Chengdu, 610000, China.; 6 Department of Experimental Research, Sichuan Clinical Research Center for Cancer, Sichuan Cancer Hospital & Institute, Sichuan Cancer Center, University of Electronic Science and Technology of China, Chengdu, 610000, China.

**Keywords:** terahertz, precision oncology, tumor imaging, cancer therapy, artificial intelligence

## Abstract

THz technology is expected to provide breakthroughs for precision oncology due to its physical nature, such as non-ionizing radiation nature, sensitivity to water and fingerprint recognition. Yet, its clinical application is severely limited due to their drawbacks: shallow penetration depth, difficult interpretation and sensitivity. This review examines recent interdisciplinary advances that integrate THz technology with materials science, nanotechnology, artificial intelligence (AI), computational modeling, gene editing, and microfluidics to develop intelligent diagnostic and therapeutic systems capable of supporting the full oncology continuum—from tumor imaging and biomarker detection to treatment monitoring and drug delivery assessment. For example, combining THz with metamaterials or nanostructures enhances sensitivity for trace-level biomarker detection; AI algorithms enable rapid, accurate interpretation of complex spectral data for automated diagnosis; and convergence with microfluidics and CRISPR-based systems has led to ultra-sensitive liquid biopsy platforms. These integrated approaches not only address existing technical barriers but also open pathways toward multifunctional theranostic systems with practical clinical utility. By fostering cross-disciplinary collaboration, THz technology can be further optimized to enable more accurate, effective, and personalized cancer care, transforming its potential from foundational research into real-world clinical impact

## 1. Introduction

The terahertz (THz) spectrum, located between the microwave and infrared regions, typically generally ranges from 0.1 to 10 THz 1, 2. Such a specific spectrum provides THz wave physical properties which we believe is suitable for precision oncology applications 3. This is advantageous because THz radiation is not ionizing: the photon energy of a THz is of the order of millielectron volts, which is important for any kind of application involving frequent monitoring or treatment modulation 4. Another advantage is that the THz waves are absorbed by water by a strong internal contrast. This is a nice biomarker to differentiate malignant vascularised lesions from healthy surrounding tissues 5. Another benefit is that THz spectrum corresponds to the characteristic vibrational and rotational bands of different biological molecules, and one can recognize their dynamic states in direct. This property can generate a unique “fingerprint spectrum” to achieve marker free recognition and quantitative analysis without external markers 6. A notable example involves ferroptosis, where transferrin delivers iron (Fe^3+^) to cells. Molecular dynamics simulations have shown that transferrin binding to Fe^3+^ is highly wavelength-dependent. Research indicates that 34.5 THz radiation inhibits iron-transferrin binding; this effect is attributed to altered molecular binding between the ions and the protein rather than a direct change in transferrin's spatial conformation 7. From these advantages, THz precision medicine has been applied to many fields in a highly unifying manner.

From the characterization of active pharmaceutical ingredients (APIs) and monitoring of drug delivery systems (DDS), it is possible to image tumor margins or directly exert a direct effect on the disease by controlling gene expression or enzyme activity 8. Essentially, it can detect tumors inside the environment, and can be found to measure molecular biomarkers without labels, and monitor response in a safe and reproducible way. It offers potential solutions for testing tumor margins intraoperatively, detecting molecular biomarkers without labels, and tracking therapeutic responses in a safe and reproducible way. Thus, it complements the important surgical task of determining tumor boundaries accurately, making THz a breakthrough technology for accurate diagnosis and treatment 9.

However, these advantages are still hard to gain a broad impact in clinical practice due to the limited penetration depth, difficult data analysis and detection sensitivity issues 10. The penetration depth of THz radiation water-based biological tissues is often lower than 1 mm, it restricts its *in vivo* use and the researchers often have to rely on *ex vivo* samples or surface images. Also, THz spectral data can be very difficult to interpret and be hard to separate small molecular features from background noise and background noise 11.

Additionally, traditional THz detection methods usually lack the sensitivity and specificity needed for trace-level biomarker detection 11, leading to interdisciplinary approaches and new research paradigms 12. Beyond these physical limitations, the integration of other technologies is also important for engineering and transformation. For instance, building a single solid, clinically certified device combining sensitive THz detector, a chip in microfluidic and an AI processing device would be challenging in miniaturization, signal cross-talk, power control, data synchronization, cost-effective manufacturing and might be more difficult than any single technical problem and will become the subject of further research and development.

**Figure 1:**
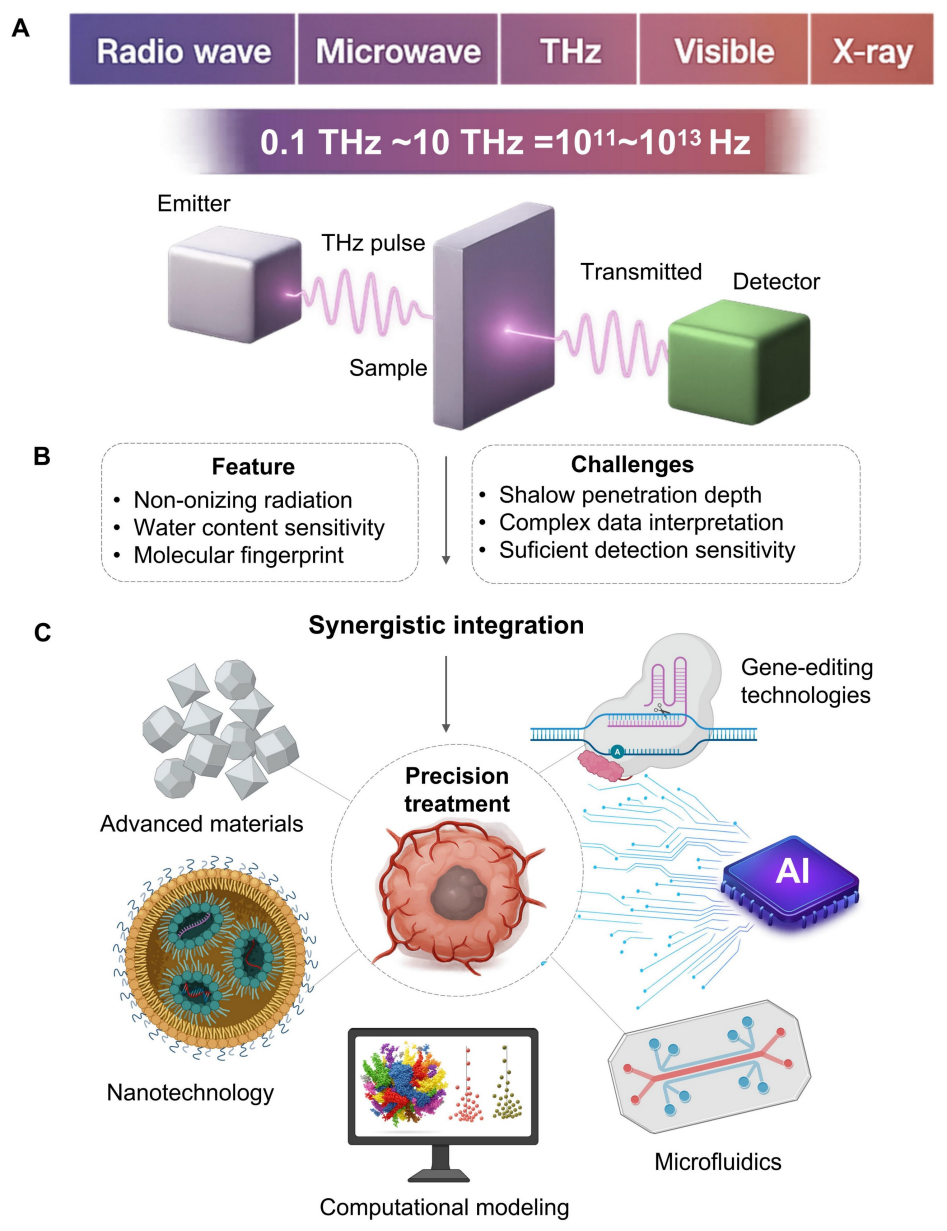
** The development direction of THz in precision oncology.** It indicates its own key, its strengths and limitations, and provides a planning strategy based on collaboration with other fields to overcome these challenges. A. THz radiation saturates the electromagnetic spectrum between 0.1 and 10 THz between microwaves and infrared light. A typical time domain system provides an emitter delivering a pulsed beam, a sample chamber, and a detector of transmitted or reflected signals. B. The technology is appealing for oncological applications (dense, water sensitive, and molecular fingerprinting). However, clinical applications are still challenging (drift tissue penetration, difficulty understanding complex data and relatively poor sensitivity to trace biomarkers). C. Pairing THz with nanotechnology, artificial intelligence, microfluidics, and gene-editing tools creates intelligent platforms. This improves detection sensitivity, decodes spectral information, automates analysis and offers targeted therapies, pushing THz to potential clinical applications.

Notably, THz technology is rapidly emerging in the field of precision oncology. We believe it holds profound implications for integrating THz systems with other emerging scientific fields, such as nanomaterials, computational frameworks, artificial intelligence, genetics, next-generation optics, and lab-on-a-chip microfluidics. This signals a departure from fundamental research toward new, adaptive diagnostic-therapeutic technologies. We believe such cross-disciplinary integration is essential for enhancing the power, discriminatory accuracy, and translational applicability of THz technology in clinical cancer care. We aim to provide concepts and methodological tools to accelerate the development of the THz medicine platform **(Figure 1)**.

## Synergy between THz Properties and Precision Oncology

Precision oncology is aimed to maximise treatment efficiency and minimize side effects by offering the proper treatment to the patient in the best time. Due to the physical properties of THz radiation, we have a high margin for safety, target recognition and noninvasive monitoring **(Figure 1)**.

### 2.1 Foundational Advantages: Safety and Intrinsic Contrast

One of the most important requirements of precision medicine is that treatments will affect tumor cells with minimal damage to healthy tissue 13. Traditional radiotherapy is ionizing and poses risk of DNA damage and secondary cancers whereas THz radiation is not ionizing 14. This risk is substantially lower, making THz-based methods suitable for repeated long-term or very detailed monitoring.

Besides safety, the technology may produce internal contrast without labels. Because THz waves are sensitive to water, cancerous tumors can be separated from healthy tissue via subtle hydration differences caused by high tumor hypervascularity and rapid growth 15. Moreover, THz radiation may probe the low-frequency collective vibrations and rotations of important biomolecules, revealing a new picture of their structure and function beyond simple water content analysis 16. This allows the ability to detect the different spectral 'fingerprints' associated to specific molecular markers or their conformational state in the tumor microenvironment that perfectly aligns with the requirement for personalized molecular-level diagnosis.

### 2.2 The THz Imaging Niche: A Comparative Perspective

The non-invasive nature of THz imaging is critical for meeting the clinical demand for real-time, minimally invasive monitoring of treatment responses. To achieve this, THz imaging must be integrated into current clinical workflows; however, this is currently hindered by its primary drawback: diffraction-limited spatial resolution. Overcoming this resolution limit is a key research challenge that is being addressed through advances in computation. Specifically, deep learning-based super-resolution methods and image reconstruction techniques leverage computational power to circumvent physical constraints. This capability is essential for making clinical imaging practical for specific applications, such as head and neck diseases 8. Hardware advances such as metasurfaces are also developing an improved sensitivity of THz detection 17. On the other hand, the collaborations between THz Imaging and nanoscale biomaterials are bringing new opportunities for early cancer detection 18. Building on these developments is an effort to develop more accessible and cheaper imaging system that is moving the technology closer to clinical implementation.

Compared to the other sources, THz imaging has a complementary role. The range of wavelengths is 30 mm - 3 mm between IR and microwave frequencies (0.1 to 10 THz), next to nearby electromagnetic frequencies 2. For instance, IR has shorter wavelengths (0,7-1000 m) and therefore larger spatial resolution, and mainly, is used to thermal imaging of surfaces 19. One clinical application of THz is to real-time intraoperative analysis of margins, especially when breast-conserving surgery is performed or non-melanoma skin cancers are removed 20. Many of these operations leave positive margins and they require costly and stressful downstream surgeries. Current frozen section pathology is slow (20-30 minutes), consumes huge amounts of resources, and analyzes only a small part of the margin.

THz radiation was more penetrative for subcutaneous structures and less sensitive to water and, therefore, was suited to study the composition of a stratified biological tissue. High-photon-energy UV radiation (10-400 nm) damaged DNA and limited *in vivo* applications to surface sterilization or fluorescence excitation 21. The non-ionizing quality of THz was a safety advantage, and the ability to recognize internal molecular signature did not require external labels. Intraoperative ultrasound is real-time and portable, but the contrast depends on the acoustic impedance, which is often small at the tumor edge 22. It is also a contact-based technique that requires coupling medium. As a non-contact technology, THz imaging is sterile and convenient.

Crucially, another unique property of THz is its own contrast mechanism and has potential for deeper detail by directly counting the higher water level and modified cell density in cancerous tissue giving a new layer of physiological data. Traditional tomography (MRI) or CT provides high resolution anatomical data 23. MRI provides excellent contrast for soft tissues but is expensive and takes long time 24. CT offers high spatial resolution but ionizing X-ray radiation is a potential health hazard 25. Positron emission tomography, usually combined with CT, provides excellent functional information but requires radioactive tracers which lead to expensive and hazardous radiation exposure 26. THz systems by comparison have typically been more portable and economical. The excellent soft tissue contrast provided by MRI/CT/PET-CT is inefficient for real-time surgical guidance due to its cost, size and scan time. New THz probes and imaging systems are becoming portable and fast enough to be suitable for a fast operating room.

Last but not least, while fluorescence imaging is highly sensitive, it is heavily dependent on extrinsic contrast agents, such as labels or probes 27. This raises additional difficulty, cost, and regulatory challenges. The main advantage of THz imaging is that its output gives intrinsic label-free contrast which simplifies the clinical process and avoids problems with probe delivery and specificity. **Table 1** illustrates the properties of THZ and other medical imaging technologies.

## 3. Synergistic THz Platforms: Integrating Advanced Technologies

A critical step toward overcoming the limitations of standalone systems is the integration of THz technology with other emerging platforms (**Table 2**). Future generations of THz matter sensing for precision cancer also require more than just the introduction of current technology, but also inspired by recent efforts of other fields to reshape the sensor-analyte interface. An effective model can be the use of highly engineered bivalent ligands in order to dimerize G protein-coupled receptors and enhance cellular signals 34. The next generation of THz biosensors could potentially be developed not just as affinity binding techniques as today. They might be rationally designed linker molecules or dynamic metasurfaces that exhibit major conformational change when a target is known leading to non-linear amplification of the THz signal.

On the same basis, progress in materials science (e.g., dipolar passivation optimized interface energetics in perovskite solar cells for dramatic performance gain 35) clearly demonstrates the critical importance of interface design. As an example, for THz sensing the idea is to design metasurfaces that do more than enhance the field. These structures require carefully tailored surface chemistries and electronic features that suppress non-specific binding while maximizing the dielectric response from target biomolecules. Achieving this level of interfacial control represents the next frontier in THz sensing, enabling highly specific and amplified signal transduction.

### 3.1 Synergy with Gold Nanoparticles

One approach reported is to rely on THz metasurfaces equipped with gold nanoparticles (AuNPs) to detect clinically significant biomarkers, like CA125 and CA199 36. The micro-fabricated sensor achieved detection accuracy of 0.01 U/mL for both markers, which outperforms previous methods and was tested on 19 clinical serum samples. This paper highlights the potential for THz immunosensors in biomarker study. This remarkable sensitivity jump is due to basic physics of plasmonics 37. When AuNPs are inserted into THz metasurface they act as nano-antennas and diffuse incoming THz radiation at nanoscale scales. There are two effects that cause a dramatic increase in sensitivity. First, the THz wave electric field may increase by several orders of magnitude at the sharp edges or “hotspots” between neighboring nanoparticles. Any biomolecules trapped in these hotspots will become strongly attached and their interaction will increase and, therefore, the measured THz signal will become much more sensitive to changes. On the second hand, AuNP maintain a localized surface plasmic resonance where the THz wave induces a collective oscillation of the nanoparticle conduction electrons 38. This oscillation is an extremely sensitive probe of the local refractive index as its resonance frequency will change with even small changes. Binding of target biomarkers to the nanoparticles surface modifies this refractive (which results in a measurable shift (Δf) in its resonance peak, turning the sensor into a very sensitive label-free refractometric detector.

### 3.2 Synergy with CRISPR/Cas12a Systems

**Figure 2:**
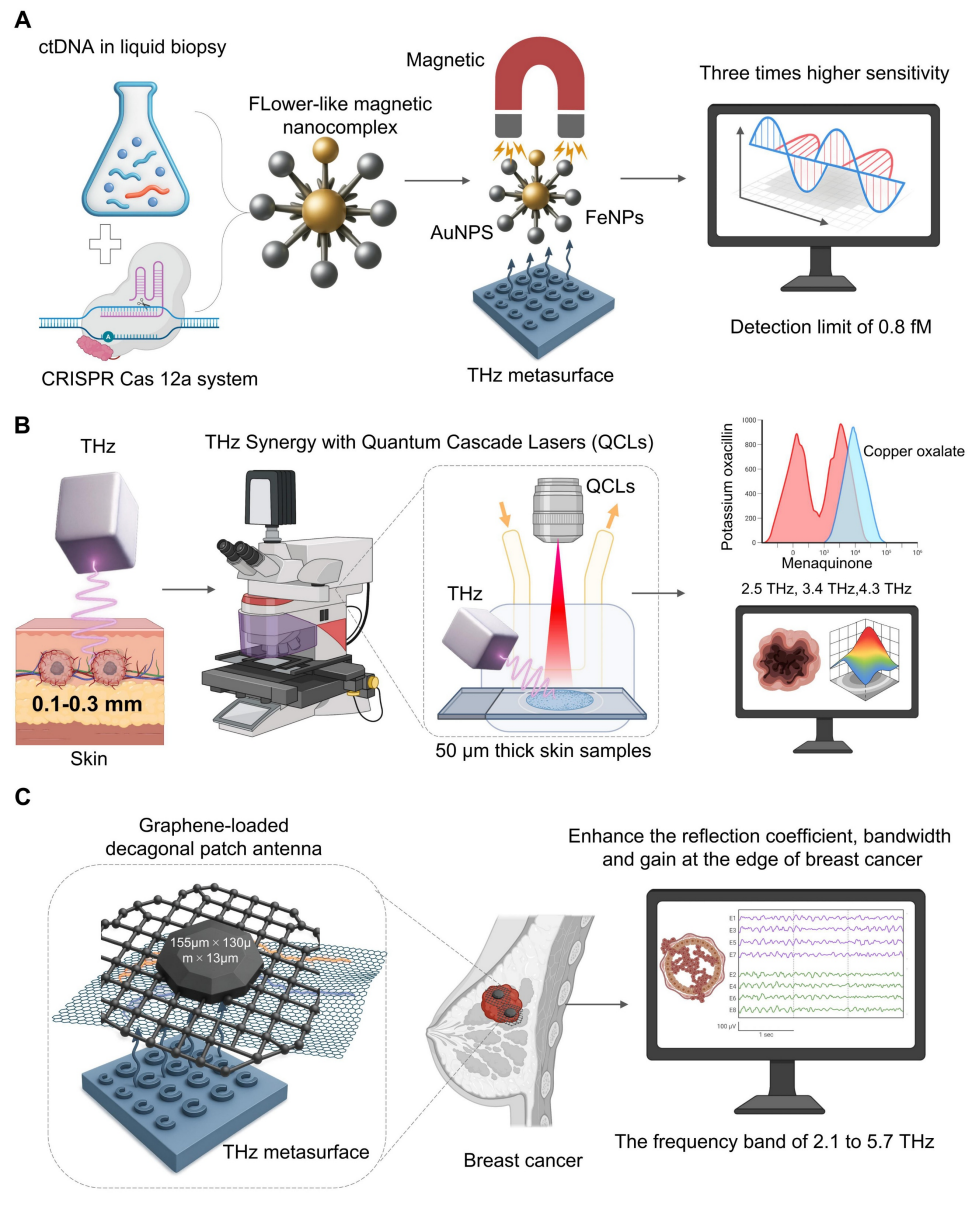
** Synergistic THz platforms to guide cancer diagnosis.** A. To detect circulating tumor (ctDNA) of CRISPR/Cas12a. On activation, the system creates a flower-like magnetic nanocomplex (auP-FeNPs). Photocribed magnetically on a THz metasurface, the complex induces strong dielectric shift, and higher THz signal sensitivity is required to measure ultrasensitive ctDNA down to 0.8 fM. B. For imaging THz shallow skin penetration (~0.1-0.3 mm), narrow band QCL source can be used for high resolution chemical imaging. Thin tissue sections (50m) are scanned by the system and some molecules (e.g. menaquinone (2.5 THz) and copper oxalate (3.4 THz), showing characteristic absorption peaks for label-free histopathology. C. In conjunction with a graphene-based decagonal patch antenna, this system enhances the local electromagnetic field. It achieves 2.1-5.7 THz performance. It delivers a better reflection coefficient, bandwidth, and gain for more sensitive and differential breast cancer detection.

A powerful liquid biopsy technique has been obtained from the combination of THz spectroscopy and CRISPR/Cas12a to allow for the sensitive and specific detection of ctDNA. In one particular study 39 (**Figure 2A**), CRISPR/Cas 12a is a mechanism for highly specific recognition. When the system binds to its target ctRNA sequence the dormant trans-cleavage is triggered. This allows the system to cut all nearby reporter probes attached to the THz metasurface. The cleavage of the probes directly changes the way the metasura interacts with THz radiation. A significant increase in sensitivity comes from starting a flower-like magnetic nanocomplex (AuNPs and FeNPs), which significantly alters the dielectric characteristics. This can be used to measure the ctDNAs, which may be measured as a measure of disease load, and we have found that the data of clinical qPCR is strongly correlated. Our platform achieved an 0.8 fM detection limit, three orders of magnitude higher than other THz biosensing. Although the direct CRISPR-THz applications in oncology are still far away, these examples provide a powerful example. Future efforts will involve expanding the range of CRISPR recognition modules and developing more advanced on-chip platforms that lead to the field of diagnostics.

### 3.3 Synergy with Quantum Cascade Lasers (QCLs)

One major drawback of traditional THz sources is the flexible multi-wavelength output of QCLs 40. For example, due to the small penetration of THz wave into skin (0.1-0.3 mm), it is appropriate for early cancer detection 41 (**Figure 2B**). A new imaging system based on narrow-band 2.8 THz QCLs was able to see different skin pathologies in 50m thick samples and match the images with histopathology and found that the method could be applied to detect sub-clinical lesions 42. With QCLs, multi-wavelength capabilities also enable accurate chemical fingerprinting which can be done beyond the single-frequency imaging. A researcher used this to clear the absorption spectra for menaquinone (2.5 THz), copper oxalate (3.4 THz) and potassium oxacillin (4.3 THz). This allowed him to identify metastatic brain tumor tissue based on its biochemical signature 43. Hence, THz platforms having QCLs capabilities offer significant potential for label-free multi-component quantitative analysis and are a major advance for diagnostic and drug analysis.

### 3.4 Synergy with Graphene

Graphene is one of the strongest materials known, featuring excellent tensile strength and mechanical stability, and is used in the medical field for orthopedic implants and dental materials 44. Graphene is high electrical conductivity and efficient for photothermal conversion, particularly for tumor therapy and biosensing 45. From THz point of view, graphene has a very distinct 2D honeycomb lattice and a tunable Dirac cone band structure, and thus is very suitable for plasmonics (**Figure 2C**). Unlike noble metals, that have fixed plasmept response, graphene's THz plasmmon resonance is electrically tunable and can be controlled by a simple gate bias, thereby being the key element of synergy. The most relevant mechanisms for improvement are tunable surface plasmons polaritons and double-resonance 46. A much stronger binding of THz wave to analytes occurs as the confined field of plasma focuses energy exactly at the interface of the graphene and analyte. The ability of adjusting the plasmonic frequency, which can be tuned for specific fingerprints may be useful for specific molecular fingerprints. New designs, such as a composite graphene-metasurface structure can produce dual-resonance mechanisms, and coupling ring-shaped graphene to an H-shaped metallic structure have achieved a significant refractive index sensitivity of 1.21 THz/RIU 47. This shows how hybrid architectures may yield new and highly sensitive resonance modes. The field is rapidly advancing, with emerging plasmonic metamaterials paving the way for on-chip integrated THz systems for intelligent information processing and communication 48. These developments in graphene and other plasmonic materials are not just enhancing sensitivity but are building the foundation for the next generation of smart, multifunctional THz biosensing platforms.

Its 2D honeycomb structure provides a high surface-to-volume ratio, enhancing local electromagnetic fields, while its dirac cone band structure allows surface plasmon resonance to cover the entire THz band (**Figure 2C**). A reported graphene-metasurface composite structure, using a dual-resonance mechanism (coupling a ring-shaped graphene with an H-shaped structure), achieved a remarkable refractive index sensitivity of 1.21 THz/RIU 47. A new type of graphene-loaded decagonal patch antenna (with dimensions of 155µm × 130µm × 13µm) can significantly enhance the reflection coefficient, bandwidth and gain at the edge of breast cancer within the frequency band of 2.1 to 5.7 THz 49. Thses design broadens the sensing window and provides a new approach for differential detection of cancer* in vitro* and in clinic.

### 3.5 Synergy with Antibody-Modified Chemical Microscopy

**Figure 3:**
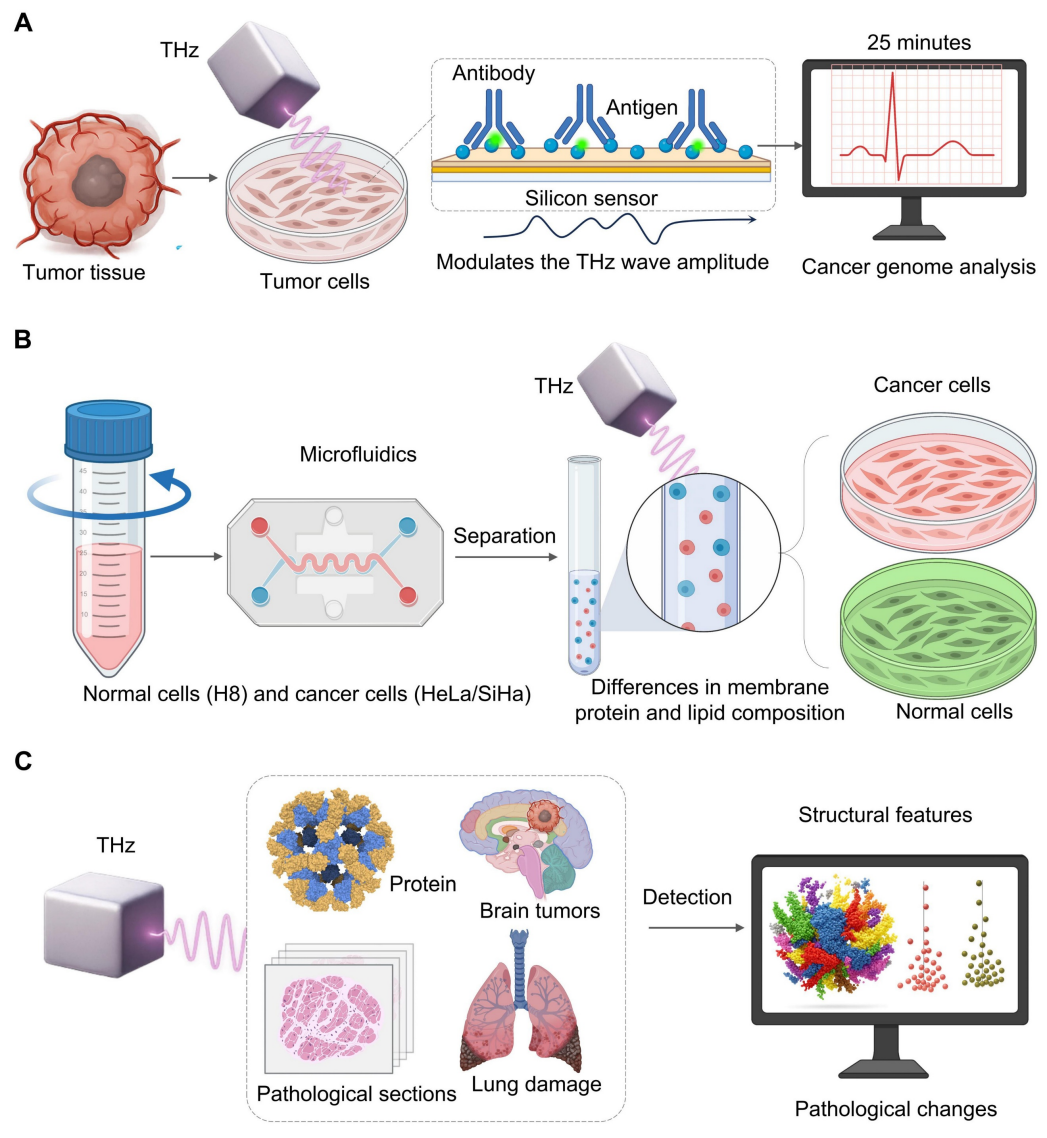
** Integrating THz technology with high accuracy and computational support.** A. Simulating cancer genome analysis based on antigen-antibody binding on a silicon sensor alters local charge density and determines THz wave amplitude. This technique computes cancer cells in less than 25 minutes using highly-processed samples and allows quick genomic diagnosis. B. Using a microfluidic chip, normal (H8) cells are first separated from cancerous (HeLa/SiHa) cells by THz spectroscopy. THz is able to discriminate them according to specific absorption signatures obtained from membrane composition, and thus, to label-free high-throughput cell characterization. C. Complex high-dimensional data from proteins, brain tumors and lung tissue are automatically processed by AI. AI algorithms detect unusual patterns and pathological features, which are not obvious to manual analysis. Such images transform raw THz data into accurate diagnoses and treatments.

As a cornerstone of personalized cancer therapy, cancer genome analysis has become a major focus of research, as understanding tumor genetics is essential for tailoring effective treatments. Evaluating the proportion of cancer cells in the sample tissue is crucial for precise genomic analysis 50. However, traditional methods take at least two days to assess an accurate genome and rely on the technical proficiency of pathologists 51. Therefore, a terahertz chemical microscope was developed. The principle relies on antigen-antibody binding altering the local charge density on the silicon sensor surfacev^52^, which in turn modulates the THz wave amplitude. The method can be completed within 25 minutes without a complex sample preparation (fixation, embedding, staining) which provides a breakthrough to quantitative analysis 53 (**Figure 3A**).

### 3.6 Synergy with Microfluidics

Microfluidics allows control of fluids at micrometer-scale that are fine-tuned for mixing, separation, and reaction 54. Small devices are portable and quickly to respond, and can be adapted for clinical diagnosis and point-of-care applications 54. When THz spectroscopy is coupled with microfluidic chips, high throughput, non-invasive and label-free analysis of cells can be performed. For example, Xiaoyue Yang *et al.* 55 investigated cervical cells with a PET-based microfluidic device. They found that in the 0.2-1.2 THz range normal cells (H8) and cancer cells (HeLa/SiHa) present different THz absorption peaks due to differences in their membrane protein and lipid content. They also show that absorption increases with cell density due to changes in their dielectric properties due to the enhanced intercellular connection, which allows for accurate sample and analysis of cell dielectrics in an environment that is well-controlled (**Figure 3B**).

### 3.7 Data Intelligence: The AI-Powered Layer

Large and complex datasets produced by modern THz platforms require a higher level of intelligence, and it is increasingly performed by artificial intelligence (AI) 56. Finding meaningful biological conclusions based on complex THz spectral data is highly dependent on AI algorithms, which range from traditional machine learning methods such as Principal Component Analysis (PCA), k-means clustering, Support Vector Machines (SVM), and Random Forests (RF) that are capable of extracting feature, segmenting, and training features to deep learning (DL) models such as Convolutional Neural Networks (CNN), Recurrent Neural Networks(RNN), Long Short Term Memory (LSTM), Generative Adversarial Networks (GAN), and Graph Neural Networks. The latter models automatically learn hierarchical patterns from raw or high dimensional data 57. The selection of AI algorithms is a function of the type of THz data that may be being analyzed, whether it is 1D spectra or 2/3D images, and to the target task (e.g., classification, segmentation or regression). When the task is 1d THz spectra, and we seek to classify samples (cancerous versus healthy) by their spectral fingerprints, traditional machine training models usually perform very well, and generally involve the introduction of feature extraction, such as PCA, to lower the dimensionality and identify important spectral differences.

When it comes to 2D THz images with high spatial information, deep models (CNNs specifically) are often preferable. Unlike previous based approaches that rely on features designed hand, CNNs learn the best hierarchical representations directly from the data of the generated pixels in an end-to-end manner which is very useful to capture cancer locations in a larger image. For the very specific and important task of semantic segmentation—i.e., of showing the precise borders of a tumor pixel by pixel—specialized CNN designs like U-Net are currently known. Jia Shi *et al.* 58 showed the potential for such a workflow to quickly classify lung tissue status by SVM and RF, showing the potential of this workflow for fast feature-based decisions.

While 2D THz imagery are highly spatially detailed, deep learning models such as CNNs have a unique advantage since compared with hand-crafted features, CNNs learn hierarchies from raw pixel data in an end-to-end way, to detect cancerous regions in rich tissue images. For the semantic segmentation task (identifying tumor boundaries at the pixel level), well-trained architectures such as U-Net offer a good performance. As indicated by Mavis Gezimati *et al.* 59, U-net's encoder-decoder can capture both deep context information and fine-grained localization, which is suitable to automatically segment brain tumor in THz images.

AI-THz are being closely related due to their desire to have fast, label-free, and objective analysis, see **Figure 3C**. Their results are also seen in other applications. Thirumuruganandham SP *et al.* 60 applied AI to a complex THz spectrum from protein simulations, detecting small stability-related conformational changes. Yim MS *et al.* 61 showed that a CNN-based model could accurately extract cancer sites from digital pathology images, suggesting that similar automatic segmentation would be applicable to THz images. These cases emphasize a key observation: THz capture data representing a sample's molecular or structural features and then the collected data are processed by a specific AI algorithm to classify, segment, or predict the problem, speeding up clinical decision making.

Although AI has made great progress in terahertz tumor biology, there are several important challenges to be addressed. One is the “black box” of deep learning models. Lack of intuitive knowledge regarding how a model gets diagnosed can confuse clinicians and complicate error identification. As such, developing and incorporating explainable AI techniques to make model decisions more transparent is necessary for validation and approval. Another major barrier to the performance of any AI model is the quality and amount of training data that the field has. In current practice, the large scale, multi-institutional and highly annotated THz oncology data set is lacking which increases the risk that models may not generalize beyond their initial training set. Designing well-known data collection procedures and shared public repositories are important for building trustworthy clinical decision-support tools. Lastly, small datasets yield high risk of overfitting, that models memorise the data from data sets but learning generalized biological patterns, thus losing good performance on training data, but not with new patients to perform well when the model has failed clinically to date. The problem must be addressed in order to ensure rigorous validation on independent test sets and regularization.

## 4. Applications of THz Technology in the Oncological Workflow

THz technology is rapidly transitioning from a laboratory experimental tool to a clinical instrument in oncology. It now encompasses the entire patient care continuum, from diagnosis and therapeutic intervention to the real-time monitoring of drug delivery systems. The application of THz in oncology dates back to 2003, when Woodward *et al.* reported the first images of human basal cell carcinoma and demonstrated a strong correlation between the detected sites and histological pathology 62. This was followed by Fitzgerald *et al.* 63 who first performed THz pulsed imaging of human breast tumors, and showed different absorption properties of normal and cancer tissues. There is significant ongoing research: Mogensen M *et al.* (2007) 64 imaged paraffin-embedded skin cancer tissues; Brun *et al.* (2010) 65 applied reflection-mode THz imaging to pancreatic tumours; Bennett DB *et al.* (2021) [66]used PEGs to treat porcine cornea with consistent and controlled water concentrations; Yiwen Sun *et al.* (2012) 67 studied the temperature dependent response of breast cancer biomarker HER2; Sim *et al.* (2013) 68 used THz images of excised oral cancer in frozen temperatures.

In the mid-2010s more technology was developed. Dibo Hou *et al.* (2014) 69 used THz spectroscopy to diagnosis gastric cancer from dehydrated human skin; Lu Rong *et al.* (2015) 70 used continuous wave THz digital holography for liver cancer tissue images; Martin *et al.* (2016) 71 used a reflection mode THz image system with 0.58 THz optically pumped gas laser for non-melanoma skin cancer and Zhaoxin Geng *et al.* (2017) 72 detected the AFP and GGT-II biomarker at 19 GHz. Applications grew with studies by Truong *et al.* (2018) 73 on breast tissue phantoms and Duan *et al.* (2019) 74 on discriminating hepatocellular carcinoma.

**Figure 4:**
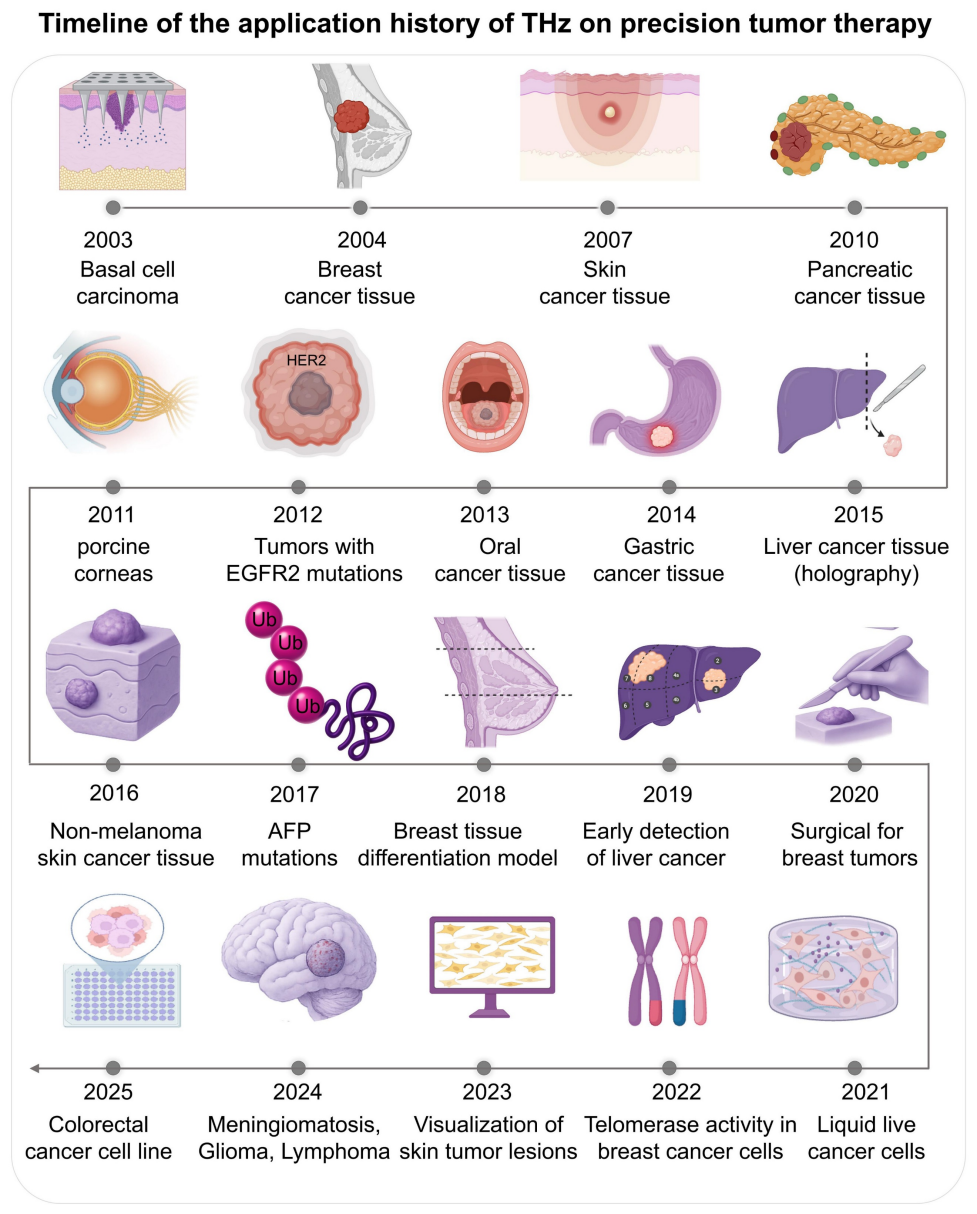
**There are key historical milestones in the application of THz technology for accurate cancer.** Starting with initial studies in imaging excised tissues (basal cell carcinoma 2003, breast tumors 2004), the next decade of studies was expanding to other cancers (pancreatic, oral, gastric), biomarkers (HER2, 2012) and imaging technologies (digital holography 2015). In recent years, clinical applications such as surgical margin monitoring (2020), liquid biopsy analysis (2021) or molecular (e.g., telomerase activity modulation) have taken the forefront. Recent developments ranging from THz to IR converters in skin lesion visualization (2023), illustrate an increasing trend toward an advanced tool integration. This show that THz has evolved from a lab curiosity to a technology with potential within the medical community.

Recent years mark a transition towards clinical utility and intelligent systems. Nagma Vohra *et al.* (2020) 75 analyzed fresh breast cancer specimens to shorten surgical margin assessment time. Yuqi Cao *et al.* (2021) 76 differentiated human colorectal cancer cell lines using THz time-domain attenuated total reflection spectroscopy. In 2022, Xianhao Wu *et al.* 77 relate THz spectra with the glioma histopathology. Kamil Moldosanov *et al.* 42. have used a THz to IR converter to capture skin tumours. Junkai *et al.* 78 find 33 THz photons which suppress telomerase activity in breast cancer cells and Nikita Gurjar *et al.* have used THz polarimetry to make image contrast better in breast cancers surgical samples. In 2025 Haiyun Ya o *et al.* 79 are developing a graphene microfluidic device to detect live cancer cells, which are discussed in Figure 4.

### 4.1 Diagnostic Imaging: From* Ex Vivo* Tissues to* In Vivo* Potential

Due to a high contrast to optical properties, THz imaging can discriminate tumor from normal tissue. In the 0.5-1.9 band, tumor tissue can absorb and refract significantly higher than other tissues (P<0.01) and contrast is high at 1.5 THz 80. In a colorectal cancer mouse model 1.8 THz is chosen as best frequency 81. Furthermore, THZ biosensors based on microfluidic chips can discriminate two cells at a sensitivity of 642.5 GHz/RIU which allows high-accuracy discrimination of tumor cells 82.

Another key breakthrough was achieved by Zihan Zhao *et al.* 83, who proposed a small THz “metachip” to detect and measure human cancer cells, with a 1-3 THz wavelength high quality factor (Q-factor) of up to 230. By analyzing its interaction to different cancer cells the device results in high-dimensional spectral features that can be converted into maps of labels to detect, quantify and measure cancer. The system was used to detect up to 15 different cancer cells with a high detection accuracy of 93.33% and quantitative sensitivity of 1320 kHz cells/mL. This low-cost, compact, and label-free device was considered to be a promising method for early detection of cancer. Pioneering studies in different cancers, such as basal cell carcinoma, breast, liver, and cervical cancer, lay the groundwork for the general field 84. The initial research progressed quickly from bulk tissue analysis to target a certain cancer biomarker and image different *ex vivo* tissues (oral and gastric cancer) in different circumstances, often using high-end techniques such as digital holography 68, 69, 85.

### 4.2 Direct Therapeutic Modalities

While the effects of the treatment are increasingly present, biological effects are still under active scientific investigation and debate. It remains to determine if these nonthermal effects are caused by resonant effects, strong field interactions, or subtle thermal effects to inform our future therapeutic plan. We discuss the main competing hypotheses from THz below.

The resonant excitation hypothesis is that certain THz frequencies directly correspond to low-frequency collective vibrations of macromolecules, for instance the conformational “beating” modes of proteins, or the lattice vibrations of DNA. This resonant energy transfer cannot change the protein function or determine which cell signaling pathway are being switched on or off; for instance, the reported frequency-dependent effect of telomerase activity (33 THz) 86 and DNA demethylation (1.6 THz) 87 can be attributed to this model, which is quite likely to be broadband and less frequency-independent. In another work, Cheon *et al.* 88 found a characteristic resonance of DNA methylation in blood cancer cells by using a THz-TDS system, with a characteristic resonant at 1.7 THz. Xionion with high-power 1.5 THz waves demethylated 10%-72% of the cancerous DNA in cancer cells, yielding a new epigenetic approach.

Another hypothesis is based on strong electric fields of high-intensity THz pulses. These fields can, from gigavolts per meter, produce large ponderomotive forces that may influence the conformation of voltage-gated channels such as the results of flux modulation. Such magnetic fields may also cause short-lived electroporation of the cell membrane, changing cell permeability and signaling without much temperature change 89. The local thermal gradient hypothesis suggest that even if the bulk temperature of a sample is not large, strong resonant absorption of THz waves by water molecules trapped in or near critical nanostructures may generate highly localized temporary temperature spikes.

Addressing this complexity remains a significant challenge. It is likely that in different situations (continuous-wave vs. pulsed radiation, frequency, cell target) each of these mechanisms may play an important role and even complement each other. Future studies must involve comprehensive spectroscopic analyses and molecular dynamics simulations to elucidate the predominant biophysical interactions. This understanding is essential for translating experimental findings into predictable and effective therapeutic strategies.

### 4.3 Characterization and Monitoring of Nanotherapeutics

THz technology has become an essential analytical tool for developing, describing, and monitoring drug delivery system (DDS). Its non-destructive, high-sensitivity, fingerprint specific characteristics are changing the way we describe materials moving from bulk analysis to nanoscale analysis.

**Figure 5:**
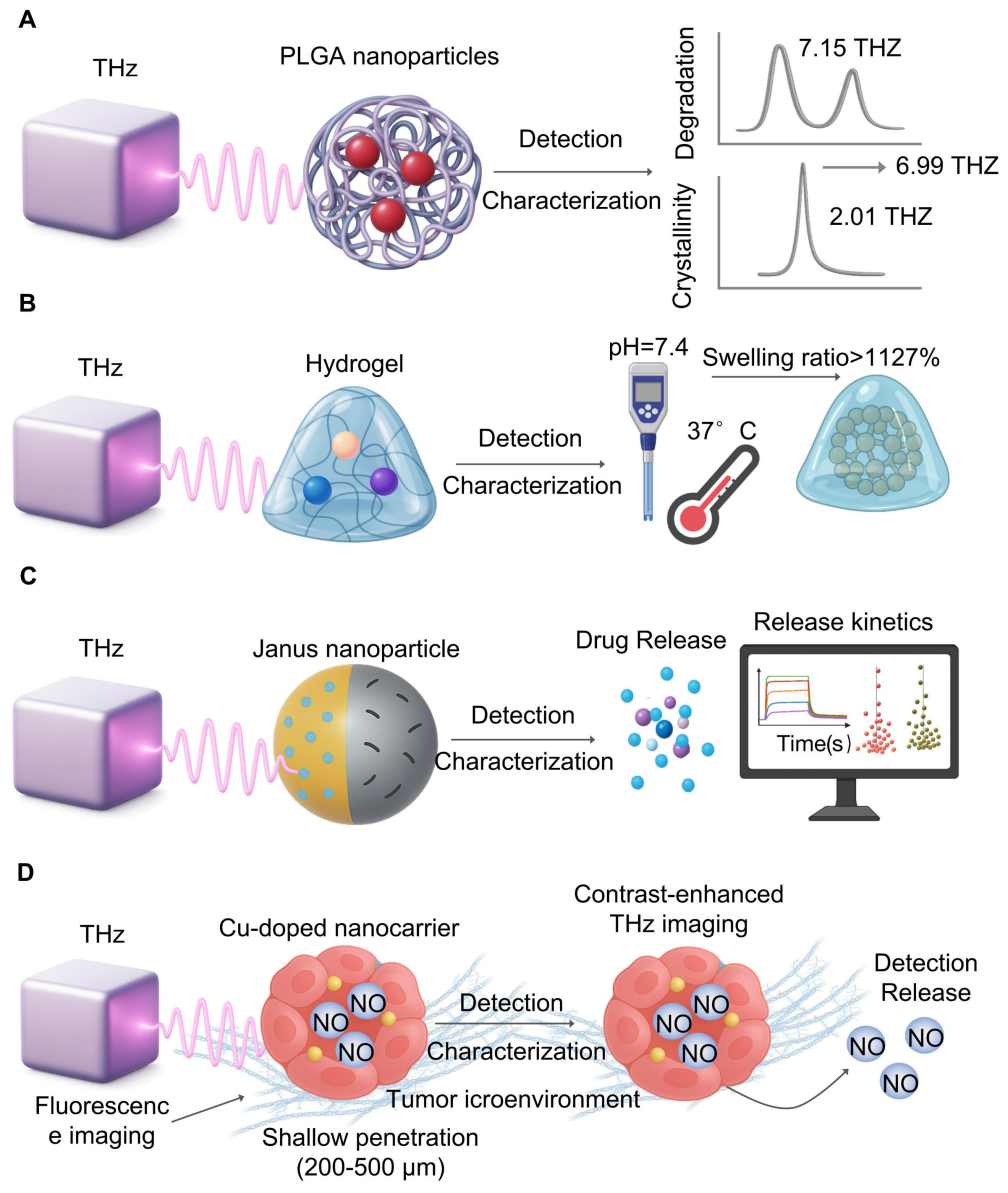
**Monitoring nanomedicines and drug delivery.** A. THz spectroscopy captures polymers like PLGA in real-time. Activity changes of characteristic peaks (7.15 and 6.99 THz) refer to hydrolysis, whereas peak intensity (2.01 THz), reflects crystallinity change, which is important for material performance. B. THZ waves are non-invasively studying hydration during the physiological conditions (pH 7.4, 37°C), thereby allowing us to measure properties such as swelling ratio, which can exceed 1127%, for drug release. C. Release kinetics of higher-level carriers (e.g., dual-responsive Janus nanoparticles) are characterized with THz. It tracks real-life payload release and provides information useful for drug delivery systems. D. THs imaging can be label-free than fluorescence microscopy, which track drug distribution in the tumor microenvironment. It is not autofluorescence, offers better penetration, and offers clearer visualization of nanocarrier behaviour.

Protein (poly(lactic-co-glycolic acid) (PLGA) is an important nanocarrier material. THz-TDS 95 (**Figure 5A**) found that, after degradation, the characteristic peak region at 7.15 THz and 6.99 THz decreases, signaling oligomerization through ester bond hydrolysis 96, an univariate relationship between the magnitude of the 2.01 THz vibration peak and the crystal of polylactic acid (PLA) allowed them to track qualitative change of conformational behaviour during material decomposition. These results suggest that THz is capable of not only detecting molecular vibration patterns when degradation, but also can perform real-time and dynamic performance evaluation based on parameter values like attenuation coefficients, offering valuable analytical guidance for designing biodegradable implants.

### 4.4 Monitoring of Drug Release Dynamics from Smart Carriers

Terahertz technology demonstrates exceptional capabilities in monitoring the *in vitro* release kinetics of stimuli-responsive carriers 97. THz-TDS provides non-invasive tracking of drug release, as evidenced by studies observing hydration dynamics in polymer-coated tablets 97. For smart hydrogels 98 (**Figure 5B**), THz spectroscopy has successfully monitored hydration changes under varying pH and temperature conditions, revealing swelling ratios reaching up to 1127% at pH 7.4 and 37°C. In the case of dual-responsive magnetic Janus nanoparticles 99 (**Figure 5C**), THz technology characterized their release profiles, showing that NIR laser-induced heating markedly accelerated doxorubicin release.

Lara Heidrich *et al.* employed terahertz time-domain spectroscopy to continuously measure the crystallization process of nifedipine, successfully characterizing crystal form changes in amorphous nifedipine over a 144-hour period. Xiang-Jun Li *et al.* (2021) 100 reported a sensing approach that leverages geometric scanning of metal metasurfaces and pseudo-surface polarization to achieve sharp resonance. This scheme demonstrated an approximately 200-fold enhancement in ultra-wideband terahertz absorption and proved capable of clearly identifying trace thin films of lactose. These examples illustrate how THz, a sensitive device due to its sensitive sensing of trace chemicals and unique hydration state detection, can be used to study release of stimuli-responsive carriers 101.

### 4.5 Imaging in the Tumor Microenvironment

Visualizing drug distributions of a tumor microenvironment is essential, and THz imaging is a label-free alternative 102 to fluorescence imaging that often suffers from low depth (200-500 nm) and autofluorescence (**Figure 5D**). Debamitra Chakraborty *et al.* 103 utilized a clinically relevant genetically engineered mouse model, conducting THz-TDS studies on paraffin-embedded pancreatic ductal adenocarcinoma samples. By extracting the terahertz refractive index and absorption coefficient from THz-TDS data, they could accurately and reproducibly reflect the physical characteristics of the tumor tissue microenvironment. Sayuri Yamaguchi *et al.* 104 achieved discrimination between normal and tumor tissues based on complex refractive index spectra acquired via reflected terahertz time-domain spectroscopy from rat glioma models. The elevated optical constants observed in tumor regions, resulting from higher cell density and water content, form the basis for this contrast. Imaging based on such tumor microenvironment characteristics has also been applied in several other glioma models 105, 106. THz imaging's intrinsic contrast capabilities present a distinct advantage for addressing challenges like tumor heterogeneity and drug penetration barriers.

### 4.6 Targeted Modulation of Ion Channels

**Figure 6:**
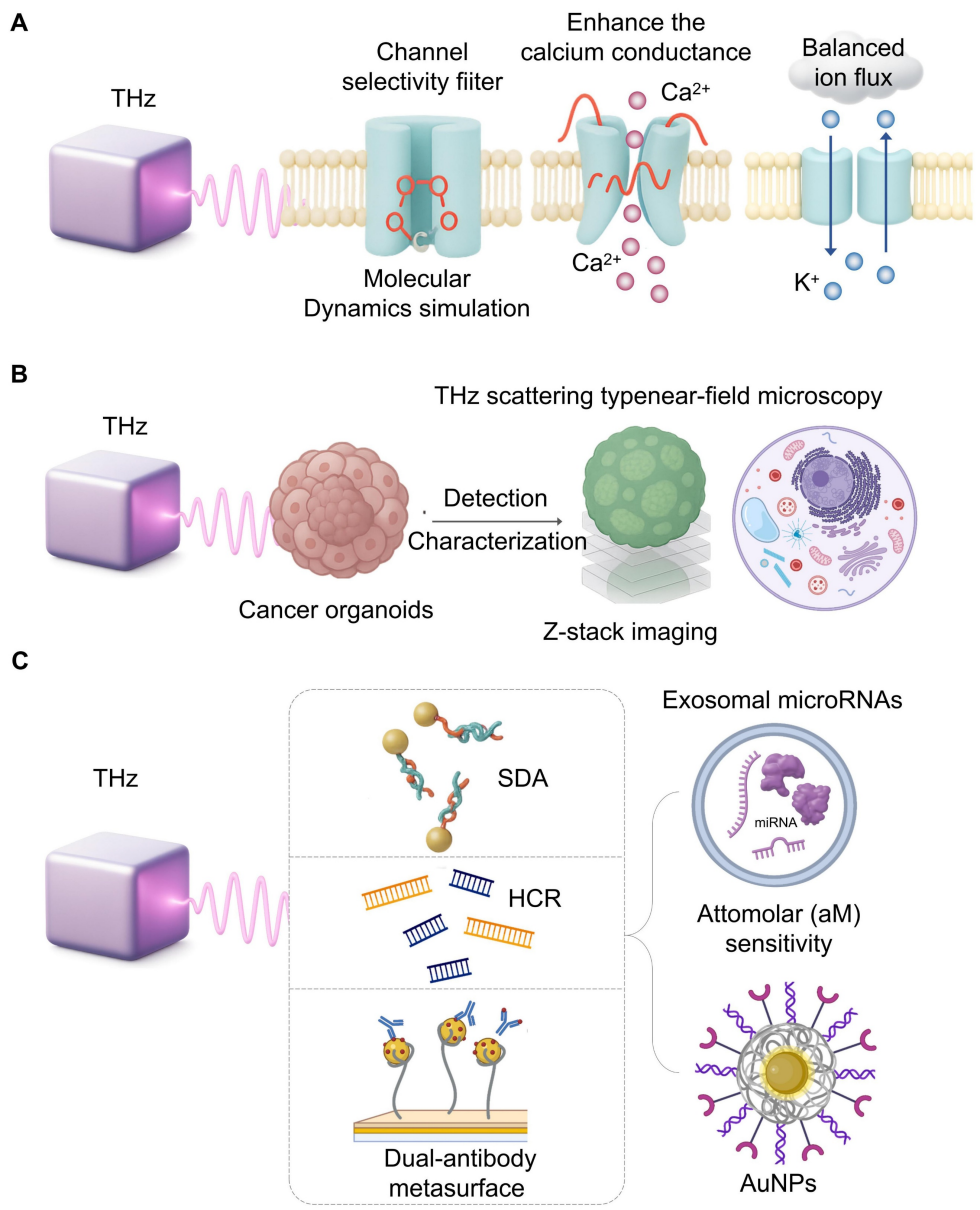
** Cellular modulation and ultrasensitive sensing with THz.** A. Molecular dynamics simulations suggest THz waves can directly interact with ion channel selectivity filters, altering their conformation. This interaction can enhance calcium conductance or rectify potassium flux imbalances, proposing a novel, non-invasive therapeutic strategy for related diseases. B. Combining THz scattering-type near-field microscopy with Z-stack imaging enables label-free, subcellular resolution analysis of cancer organoids. This approach provides detailed structural information for high-throughput, preclinical drug screening without fluorescent labels. C. Achieving the attomolar sensitivity required for liquid biopsy involves coupling THz biosensors with signal amplification strategies. Techniques like strand displacement amplification (SDA), hybridization chain reaction (HCR) with AuNPs, and dual-antibody metasurfaces empower ultrasensitive detection of specific microRNAs.

The interactions between THz waves and ion channels at the nanoscale is a topic of study. Molecular dynamics show that THz wave may sing to the symmetric vibrational frequencies of a channel selectivity filter (C=O bond stretching) which will alter its shape and ion flux 107. For example, 42.5 THz waves have been shown to enhance the calcium conductance of the Ca(v)1.2 channel 108. Similarly, mutations in the KCNA2 gene (encoding the Kv1.2 subunit) can cause neuronal hyperexcitability and epilepsy; THz waves, by modulating Kv1.2 permeability, may offer a way to correct this ion flux imbalance 109. THz radiation (51.87 THz) can increase the flux of potassium ion (K⁺) channels and affect the electrophysiological characteristics of tumor cells 110 (**Figure 6A**).

34.5 THz radiation can specifically inhibit transferrin (Tf) -bound Fe^3+^ in A549 cells, reducing intracellular iron levels, lipid peroxidation and cell death 7. In *Geobacillus icigianus* cells 111, the homeostasis system of transition metals such as copper and zinc is sensitive to THz radiation, and the expression of related genes is significantly downregulated 10 minutes after radiation. Therefore, THz can affect the chaperone proteins in the cellular transcription process by regulating the above-mentioned metal ions. Although the research on the regulation of copper and zinc in tumor cells by terahertz has not been studied at present, the exploration in this direction in the future may be a new one.

### 4.7 High-Throughput Drug Screening with Organoids

Cancer organoids, which recapitulate tumor architecture, are revolutionizing preclinical drug screening 112. While Z-stack imaging with fluorescence is a common high-throughput method, THz scattering-type near-field microscopy offers a path to label-free, subcellular resolution 113 (**Figure 6B**). Its ability to clearly resolve chloroplast structures on a gold substrate demonstrates its potential to provide detailed structural information within organoids without fluorescent tags. In one study 114, Z-stack imaging technology was used for the imaging of three-dimensional organoids to collect the maximum cross-sections of all organoids in a single imaging. A high-throughput organoid evaluation system for drug screening was developed by integrating Z-stack imaging with fluorescence labeling. This system employed patient-derived colorectal cancer tissues to generate four organoid phenotypes with defined sensitivities to 5-fluorouracil (5-FU-S/R) and CPT11 (CPT11-S/R). Subsequently, the system was applied to analyze treatment responses, quantifying organoid viability post radiotherapy or chemotherapy through calcetin live-cell staining and ImageJ analysis. Furthermore, according to reports, by combining THz scattering near-field imaging technology, THz can break through the diffraction limit of traditional far-field imaging, thereby achieving clear identification of chloroplast structures on gold substrates at the organoid level 115.

### 4.8 Ultrasensitive Detection of Exosomal miRNAs

Exosomal microRNAs (miRNAs) are key liquid biopsy biomarkers, but their detection is challenging due to low abundance and high homology 116. The intrinsic resonance of nucleic acids in the THz range makes THz biosensing ideal for this task. To achieve the required sensitivity, THz metamaterials are coupled with signal amplification techniques. One approach for pancreatic cancer detection integrated strand displacement amplification, achieving attomolar level sensitivity for a panel of exosomal miRNAs 117 **(Figure 6C)**. Another lung cancer platform combined a hybridization chain reaction with AuNPs 118. A gastric cancer platform used a dual-antibody metasurface as exosomes and a detector with a detection limit of 1/10/mL 119. These high-quality biosensors show how THz can transform liquid biopsy.

## 5. Challenges and Future Perspectives

Although THz has tremendous potential, its path from the lab to general clinical use is full of challenges that will affect the future of the field (as exhibited in** Table 3**).

### 5.1 Technological Bottlenecks

The main physical problem is the shallow penetration of THz waves into water (around 300m in human skin) that, at present, only surface or *ex vivo* studies can perform. However, how to take advantage of THs to control and study traditional Chinese medicine in cancer is not entirely open 120. The tremendous potential of THzes may lead to new discoveries of mechanistic medicine.

### 5.2 Biosafety Concerns

While the THz radiation is not infectious, its bio-effects are not a binary problem, but rather a new topic of research with seemingly contradictory results. In general, the lower frequency (2 THz) with low power continuous wave sources, as it is typically the case in many diagnostic imaging and spectroscopy applications, is considered to have a higher biosafety profile, and there has been a small or no genotoxic effect when it is applied. For example, a study for adult fibroblasts with 0.15 THz has no significant damage to genome 121, so the range of this window is likely to be safer. On the other hand, biological effects become more sophisticated at higher frequency or at high intensity pulsed sources, where we have “dual-sources” of THz bio- effects: irradiation at 33 THz shows therapeutic effect, suppresses cancer cell tumorigenicity by suppressing telomerase activity 86 with high intensity pulses (e.g. a peak intensity of 21 GW/cm^2^) and some of these studies show no increase in DNA damage markers like γH2AX in CNS tumor cells, suggesting a potentially therapeutic window 89. These results are exciting from the therapeutic perspective, but also pose safety concerns that need to be carefully described. They emphasize the need for dose-response studies to determine the proper distance between therapy benefit and unacceptable toxicity. Different cell types differ dramatically in their sensitivity to THz sources **Table 2**. Perhaps the most critical gap in our current understanding is the lack of comprehensive long-term *in vivo* safety studies. Most studies have been performed on cultures of cells, which cannot capture entire organism's complex systemic response. Designing clear evidence-based safety limits for acute and chronic exposure to preclinical animal models is a critical prerequisite for developing any therapeutic THz application to human clinical trials.

### 5.3 Challenges in Clinical Implementation and Equipment

Commercial THz systems can be mainly classified as time domain spectroscopy (TDS) or frequency domain spectroopy (FDS) that excited a sample with ultrashort THz pulses and measured the temporal response, providing broadband spectral coverage. TDS is a non-destructive and flexible system that may have long acquisition times and high-power sources 122. FDS, on the other hand, relies on continuous-wave frequency tunable sources, and is able to produce narrow bands which allow good spectral access, but is less sensitive to the environment and requires accurate, high quality optics 123.

In addition to the aforementioned aspects, it is important that smart, low-cost and easy-to-use instruments are available for translation. It is highly expensive and time-consuming for new multi-modal diagnostic or therapeutic devices to be approved by a physician. It can also be said that if a device is to be adopted by a clinician it is a device with a simple user interface that performs complex calibration and analysis. We claim that a scientific system requiring a PhD in physics to run will never be used by the clinician by its technical facilities.

## 6. Conclusion and Future Perspective

### 6.1 Prioritizing Biosafety and Mechanistic Understanding

Before complex applications can be widely implemented, fundamental questions regarding biosafety and interaction mechanisms must be resolved. The most immediate priority is the collaborative establishment of standardized biosafety protocols, which should include consistent dosimetry, exposure parameters (frequency, power, duration), and defined biological endpoints for rigorous long-term *in vitro* assessments. This approach is essential to clarify safety guidelines and overcome the prevailing inconsistencies in current *in vitro* findings.

Parallel to safety assessments, gaining a deeper understanding of key biophysical interactions is essential. Research efforts must be expanded utilizing advanced multimodal approaches—such as THz spectroscopy combined with Raman microscopy or molecular dynamics simulations—to fully integrate and characterize resonant, electric field, and localized thermal effects on biological systems. Elucidating the underlying mechanisms of these effects is a critical prerequisite for harnessing them effectively in therapeutic applications.

### 6.2 From Benchtop to Bedside: Engineering Practical Systems

With more knowledge, we need to develop clinically useful platforms. The key task is to bridge the “benchtop-to-bedside” gap by building integrated multi-modal systems, and where future success should be measured not only by laboratory sensitivity, but also by developing robust, low-cost, and flexible platforms that integrate into existing clinical workflow. This requires a collaborative, interdisciplinary effort between physicists, engineers, data scientists, and clinicians to design the next generation of THz platforms.

### 6.3 Building Trustworthy AI with Robust Data

Large, diverse and annotated THz oncology data needs to be provided by equally smart analytics. For the development of clinically successful AI systems we propose multi-center AI datasets. The biggest bottleneck in building clinically powerful AI systems is the lack of large, diverse, and annotating THz datasets. We propose shared FAIR data platforms to train and validate models at a scale that lowers institutional bias and overfitting, paving the way for approved clinical diagnosis and prognostic algorithms.

### 6.4 Charting New Frontiers in Diagnosis and Therapy

With a level of safety, mechanistic understanding, and tools, we hope to experiment with new applications. Future research should address precisely modeling THz bio-effects to leverage them for treatment, and may be used in regenerative medicine and evidence-based research on traditional therapies. Finally, and by offering an opportunity to illuminate the molecular and cellular dynamics of cancer, THz is a source of insight-not only that but it offers the tools to change the treatment process-make cancer treatment more accurate, effective and personalized.

## Figures and Tables

**Table 1:** Comparative Analysis of THz and Other Clinical Imaging Modalities

Index	THz	IR	UV	Ultrasound	CT	X-ray	PET-CT	Fluorescence Imaging
Penetration Depth	<1 mm (*in vivo*, aqueous tissue); up to several cm (*ex vivo*, dehydrated/low-water content samples)	< 1 mm	< 0.1 mm	Several cm	Whole body	Whole body	Whole body	Wavelength-dependent (<1 mm to several cm)
Detection Time	Milliseconds	Milliseconds	Milliseconds	Minutes	Minutes	Seconds	Hours (incl. uptake)	Seconds
Frequency/ Wavelength	Freq: 0.1-10 THz WL: 30 µm-3 mm	Freq: 300 GHz-400 THz WL: 0.7-1 mm	Freq: 0.75-30PHz WL: 10-400 nm	1-20 MHz	100 kHz-100 MHz	10¹⁶-10¹⁹ Hz WL: 0.01-10 nm	511 keV γ-ray	400-1700 nm
Specificity/ Accuracy	Medium (molecular signatures)	Medium (thermal)	High (surface)	Low (structural)	Medium (density)	High (dense tissue)	High (metabolic)	High (probes)
Key Features	Non-ionizing; Water-sensitive; Fingerprint spectra	High resolution; Thermal imaging 27	High energy (DNA risk); Surface use 28	Real-time; Requires coupling agent 29	High anatomical resolution; Ionizing 30 radiation	Standard for bone/lung; Ionizing radiation 31	Functional imaging; Requires radiotracer 32	High specificity; Requires extrinsic probes 33

**Table 2:** The therapeutic effects of terahertz on different tumor types

Working Freq.	Irradiation Time	Tumor Type	Therapeutic Outcome	Refs
33 THz	21 days	Breast Cancer (4T1)	70% reduction in tumorigenicity; suppresses telomerase activity.	79
1.65 THz	N/A	Breast, Colon, Skin Cancer	Ablation of tumor tissues.	90
1.5 THz	N/A	Laryngeal Carcinoma	Absorption coefficient highly correlated with cancer cell nuclei proportion (r=0.971).	68
0.1-3 THz (21 GW/cm²)	30 min	Neuroblastoma (SK-N-BE(2))	Differential effects on SK-N-BE(2) vs. non-tumor neural progenitor cells.	89
0.2-1.4 THz	N/A	Glioma	Significant difference in THz absorption between normal brain, tumor-adjacent tissue, and tumor core.	77
0.2-1.4 THz	N/A	Human Colon Cancer (DLD-1, HT-29)	Random forests (RF) could discriminate cancer cells from normal ones.	76
83 THz	N/A	Colon Cancer (HCT-116)	Inhibited cell migration by 50% and glycolysis by 60% via altered chromatin accessibility.	91
0.46 THz(600 mW/cm²)	30-60 min	Cervical Cancer (HeLa)	Disrupted cytoskeleton integrity, inhibiting cell division; high-power pulses also inhibited glycolysis.	92
0.6-1.8 THz	30 min	Glioblastoma (U87)	Increased bound water in serum, reflecting abnormal tumor metabolism.	93
0.1-2.7 THz	N/A	Basal Cell Carcinoma	Differentiated BCC from normal tissue via moisture/vibration differences; defines pre-op margins.	62
0.14 THz	N/A	Lymphoma, Breast (MCF7), Prostate (LNCaP) cells	Varied sensitivity: LNCaP (highly sensitive, 63% survival), Ramos (moderately sensitive, 75% survival), MCF7 (unaffected).	94

**Table 3:** Summary of challenges facing THz technology in precision oncology

Challenge Category	Specific Limitation	Consequence / Impact
Physical & Technical Limitations	Shallow Penetration Depth: Extremely limited penetration in aqueous biological tissues (e.g., ~300 µm in human skin), typically less than 1 mm.	Primarily confines applications to *ex vivo* analysis or superficial tissues (like skin), hindering deep-tissue imaging *in vivo*. Acts as the primary physical barrier for the technology's clinical translation.
	Insufficient Detection Sensitivity: Conventional THz detection schemes often lack the sensitivity required for trace-level biomarkers.	Makes it difficult to detect low-abundance molecules crucial for early cancer diagnosis, limiting its potential in ultra-early screening. Requires synergistic integration with nanotechnology, AI to overcome.
Data Interpretation & Analysis	High Complexity of Spectral Data: THz spectra are rich in information but signals are often weak and easily confounded by background noise and environmental factors (e.g., humidity, temperature).	It is challenging to extract definitive biological insights from the complex signals. Creates a high dependency on advanced data processing algorithms and models, increasing the technical barrier.
Biosafety Concerns	Non-thermal Biological Effects: Despite being non-ionizing, THz radiation can induce non-thermal effects, such as altering cell membrane permeability, cytoskeleton rearrangement, and even DNA damage (e.g., H2AX phosphorylation) with prolonged or high-intensity exposure.	Raises concerns about long-term safety, especially for frequent or therapeutic use. Necessitates the establishment of clear, cell-type-specific safety thresholds and dosage standards.
	Dual Nature of Bio-effects: Different cell types show significant variations in sensitivity to THz radiation; low intensity may be beneficial (e.g., wound healing) while high intensity is harmful.	Adds complexity to clinical translation, as safety and therapeutic windows may need to be personalized. Demands precise control over the radiation dosimetry.
Clinical Implementation & Equipment	Limitations of Existing Systems: Commercial systems (e.g., TDS & FDS) have drawbacks. TDS is time-consuming and requires high-power sources; FDS is sensitive to environmental factors and requires high-quality components.	Current equipment can be slow, expensive, and not robust enough for typical clinical settings. The robustness and speed of the equipment become a bottleneck for widespread use.
	Lack of Integrated & User-friendly Instruments: There is a shortage of robust, cost-effective, and user-friendly instruments that can be seamlessly integrated into existing clinical workflows.	High cost and operational complexity hinder widespread adoption. Makes it difficult for the technology to move from specialized research labs to routine clinical departments in hospitals.
